# Reversible Optogenetic Control of Subcellular Protein Localization in a Live Vertebrate Embryo

**DOI:** 10.1016/j.devcel.2015.12.011

**Published:** 2016-01-11

**Authors:** Clare E. Buckley, Rachel E. Moore, Anna Reade, Anna R. Goldberg, Orion D. Weiner, Jonathan D.W. Clarke

**Affiliations:** 1 MRC Centre for Developmental Neurobiology, King’s College London, London SE1 1UL, UK; 2 Cardiovascular Research Institute, University of California, San Francisco, San Francisco, CA 94158-9001, USA; 3 Department of Biochemistry and Biophysics, University of California, San Francisco, San Francisco, CA 94158-2517, USA

**Keywords:** optogenetics, phytochrome, zebrafish, Pard3, asymmetric inheritance, apico-basal polarity

## Abstract

We demonstrate the utility of the phytochrome system to rapidly and reversibly recruit proteins to specific subcellular regions within specific cells in a living vertebrate embryo. Light-induced heterodimerization using the phytochrome system has previously been used as a powerful tool to dissect signaling pathways for single cells in culture but has not previously been used to reversibly manipulate the precise subcellular location of proteins in multicellular organisms. Here we report the experimental conditions necessary to use this system to manipulate proteins in vivo. As proof of principle, we demonstrate that we can manipulate the localization of the apical polarity protein Pard3 with high temporal and spatial precision in both the neural tube and the embryo’s enveloping layer epithelium. Our optimizations of optogenetic component expression and chromophore purification and delivery should significantly lower the barrier for establishing this powerful optogenetic system in other multicellular organisms.

## Highlights

• The phytochrome system has been optimized for use within multicellular organisms
• Protein recruitment can be tightly controlled to a specific subcellular region
• Protein recruitment occurs with high binding and reversal kinetics
• The subcellular localization of the apical polarity protein Pard3 is manipulated

## Graphical Abstract

**Figure:**
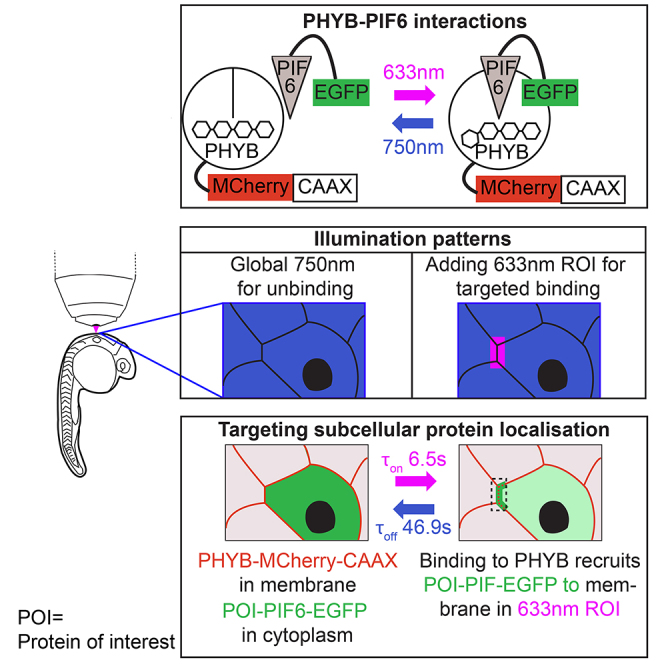

## Teaser

The phytochrome system is a powerful tool that is used to manipulate protein localization. Here, Buckley et al. optimize the phytochrome system in live zebrafish embryos and demonstrate rapid and reversible protein recruitment to specific subcellular regions within specific cells, at a high spatial and temporal resolution.

## Introduction

While gene knockout, overexpression, and mutation have been used to reveal the involvement of proteins in biological systems, more subtle manipulation of proteins is needed to interrogate their precise roles. For example, manipulation of protein localization within cells will be critical for understanding how intracellular asymmetries and polarity are established. The use of optogenetics to precisely control cell processes such as gene transcription, signaling activation, and protein dimerization has begun to transform biomedical research. It allows researchers to probe specific components of signaling pathways and cellular function, often with subcellular resolution and rapid temporal control.

The main optogenetic systems have been comprehensively reviewed elsewhere (Pathak et al., 2013, Tischer and Weiner, 2014, Zhang and Cui, 2015). Each system has a different combination of properties such as reversibility, dynamic range, and chromophore requirement (Tischer and Weiner, 2014) that must be considered when choosing which system to use in living organisms. Light-oxygen voltage (LOV)-based strategies have so far had the most success of the optogenetic systems in transferring from cell culture to whole organisms (Motta-Mena et al., 2014, Wang et al., 2010, Wu et al., 2009, Yoo et al., 2010) and, after optimization, are highly adaptable, particularly for transcriptional regulation. For example, allosteric LOV-based photoactivation of Rac1 (Wu et al., 2009) was successfully replicated in zebrafish embryos, where it was used to control the protrusion and migration of zebrafish neutrophils (Yoo et al., 2010) and in *Drosophila* ovary border cells to control collective migration (Wang et al., 2010). The cryptochrome system (Kennedy et al., 2010) has also been used in vertebrates for regulation of protein transcription, both in whole homogenates of zebrafish embryos and in the mouse cortex (Konermann et al., 2013, Liu et al., 2012).

When the focus of an experiment is to precisely control the spatiotemporal dynamics of a protein, rather than to directly alter its transcription, then arguably the most important property of an optogenetics system is its speed of reversibility. Only two optogenetics systems can be actively reversed. The first is the photoactivatable protein Dronpa, which has been used in multicellular organisms for photoswitching experiments (Aramaki and Hatta, 2006). Although successfully used in cell culture (Zhou et al., 2012), Dronpa has so far not been used to regulate protein interactions in multicellular organisms. A faster reversal can be achieved with the *Arabidopsis* red light-inducible phytochrome (PHYB-PIF) system, which comprises the phytochrome B (PHYB) protein and the basic-helix-loop-helix (bHLH) transcription factor phytochrome interaction factor (PIF; PIF3 or PIF6). These two domains are induced to bind under far-red light and the binding is reversed within seconds of exposure to infrared light but is otherwise stable for hours in the dark (Ni et al., 1999). The phytochrome system has a 10–100× larger dynamic range (respectively) than the cryptochrome and LOV-based systems (Pathak et al., 2014), and the affinity of its light-gated interaction is 100× tighter than Dronpa (Levskaya et al., 2009, Zhou et al., 2012). The phytochrome system therefore offers the highest level of spatiotemporal control of the currently available systems. An added advantage is that the wavelengths required for photomodulation (red and infrared) are far from the wavelengths of the fluorescent proteins commonly used for imaging cells and subcellular compartments. In addition, only a low light intensity is required, thus reducing the potential for phototoxicity. The phytochrome system has been highly successful in modulating signaling in single cells, such as testing the subcellular spatial sufficiency of Rac for directed cell migration (Levskaya et al., 2009) and dynamically controlling the activation and inactivation of signaling pathways in yeast (Yang et al., 2013). The tunability of this system has been used to initiate transient versus sustained Ras activation and to perform dose-response curves for isolated signaling modules in single cells (Toettcher et al., 2013). In addition, the phytochrome system is compatible with active feedback control to maintain precise signal input, despite variations in expression of the optogenetic components, or to maintain a fixed input despite cellular feedback (Hoeller et al., 2014, Toettcher et al., 2011b).

## Design

While optogenetic experimental approaches have had widespread success in cell culture and in single-celled organisms, there is a need to adapt current technologies to multicellular organisms such as vertebrate embryos. Due to its high level of control, the phytochrome system would be an optimal system for dissecting the complex signaling networks present in whole multicellular organisms and would be particularly applicable to studies of polarity, where precise spatiotemporal control is key. A recent study demonstrated nuclear protein import in superficially located cells of zebrafish embryos using whole-embryo illumination to activate the PHYB-PIF3 system (Beyer et al., 2015). However, so far the phytochrome system has not been used in multicellular organisms for fine-scale spatiotemporal or reversible control of protein localization. This is partly because, unlike the cryptochrome and LOV-based systems, which use ubiquitously occurring flavin as a chromophore, the phytochrome system requires an external chromophore, phycocyanobilin (PCB). External exposure to PCB is sufficient to mediate binding of PCB in mammalian cell culture (Levskaya et al., 2009). However, while the Beyer et al. study shows that bathing whole zebrafish embryos in PCB allows the phytochrome system to be globally used in superficially located cells, it does not allow for manipulation of cells deeper within the tissue. In the current study we have extended the use of the phytochrome system in multicellular organisms. We optimized the PHYB-PIF6 system for robust expression and optogenetic control within the zebrafish embryo. We also purified PCB and delivered it to cells deep within the developing zebrafish embryo without toxic effects. This has allowed us to demonstrate rapid binding and reversal kinetics between PHYB and PIF6 at precisely targeted subcellular regions within specific cells located both superficially and deep within the zebrafish embryo. As proof of principle within the embryo, we mislocalized the apical polarity protein Partitioning defective 3 (Pard3) subcellularly and demonstrated spatiotemporal control of activity. Our optimizations of PCB and PHYB, as well as the methodology described here, will facilitate the use of the phytochrome system in other organisms.

## Results

### Successful PHYB Expression and PCB Delivery in Zebrafish

**Figure 1:**
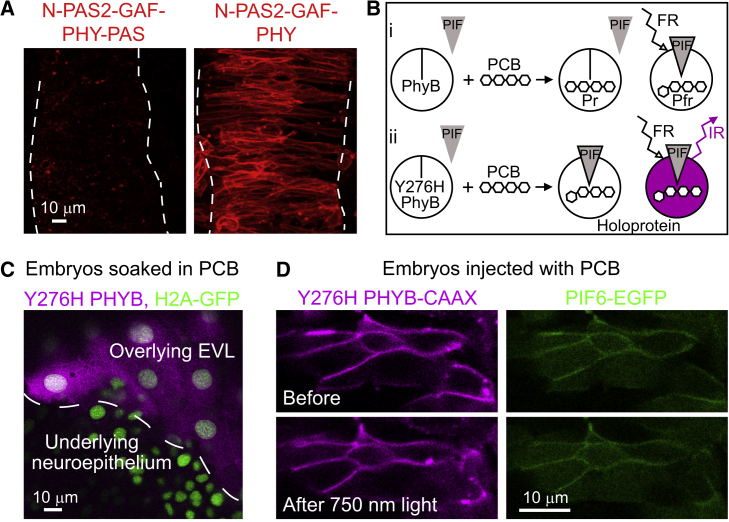
Adapting the Phytochrome System for Use in a Vertebrate Embryo (A) 50-μm z projections through the hindbrains of 14-somite zebrafish embryos labeled with PHYB-MCherry-CAAX fusion proteins with different PHYB truncations. Dotted lines denote basal edges. N-PAS2-GAF-PHY-PAS contains a PAS domain at the C terminus and was not successfully expressed in six of six embryos. N-PAS2-GAF-PHY does not contain a PAS domain at the C terminus and was robustly expressed in cell membranes in nine of nine embryos. (B) (i) Normal PHYB covalently binds PCB chromophore. Energy from far-red (FR) light causes photoisomerization of PCB and the allosteric transition of PHYB from its inactive (Pr) to its active (Pfr) state. This is reversible by infrared (IR) light exposure (not shown). The Pfr state can bind PIF. (ii) Conjugation of PCB with Y276H mutant PHYB creates an activated holoprotein that can directly bind PIF, without the need for far-red light illumination (Su and Lagarias, 2007). Energy from far-red illumination causes infrared fluorescence. (C) An oblique confocal slice through the overlying EVL and the underlying neuroepithelium of a 14 hpf embryo, labeled with Y276H PHYB and H2A-GFP and bathed in PCB. Only the EVL fluoresces under far-red light (magenta), demonstrating binding of PCB to Y276H PHYB. (D) A horizontal confocal slice through the neuroepithelium of an 18 hpf embryo, labeled with Y276H PHYB-CAAX and PIF6-EGFP and injected with PCB. Anterior is up. The membranes of all labeled cells fluoresced under far-red light, demonstrating binding of PCB to Y276H PHYB-CAAX. PIF6-EGFP was also recruited to the membrane in these cells. PIF6-EGFP recruitment to the membrane was not reversed after 5 min exposure to 750 nm light. See also related Figure S1.

We first determined how to efficiently express the photoreversible version of the plant PHYB protein in zebrafish embryos. Previous unpublished attempts found that while genetically inducing zebrafish cells to express *Arabidopsis* PIF6 protein worked well, the expression of PHYB was problematic, despite these same components working well in cell culture (Levskaya et al., 2009). To overcome this problem we found it was necessary to truncate the C-terminal end of the PHYB protein by removing both its PAS and histidine kinase-related domains (HKRDs). This truncated PHYB is robustly and reproducibly expressed in zebrafish tissue (Figure 1A).

The PCB chromophore must be bound to the PHYB protein to generate red light-dependent binding of PHYB and PIF domains (Figure 1Bi). PCB is not present in eukaryotic cells and must be added before the system will function. To test how efficiently PCB can be delivered to cells in the zebrafish embryo, we took advantage of a PCB biosensor, the Y276H constitutively active mutant of PHYB, which fluoresces under far-red light when bound to PCB (Levskaya et al., 2009, Su and Lagarias, 2007) (Figure 1Bii). We generated a version of this mutant without the C-terminal HKRD or PAS repeated domain. Embryos expressing Y276H PHYB and nuclear-labeling H2A-GFP were dechorionated at 4 hr post-fertilization (hpf) and exposed to 300 μM high-performance liquid chromatography (HPLC)-purified PCB (Figure S1 and Supplemental Experimental Procedures) in medium overnight. When imaged under 633 nm light, the enveloping layer (EVL) cells of the embryo exhibited strong fluorescence, indicating that PCB had bound to Y276H PHYB successfully. However, the underlying neuroepithelial (NE) cells did not show evidence of PCB binding (Figure 1C), showing that PCB does not penetrate beyond the first layer of zebrafish tissue. We therefore injected 1.5 pM HPLC-purified PCB directly into one cell of 16-cell stage embryos, along with the RNA encoding CAAX-linked Y276H PHYB and PIF6-EGFP. Embryos were then imaged under 633 nm light. Cell membranes of NE cells fluoresced brightly throughout the depth of the neural rod, indicating that PCB had bound successfully to Y276H PHYB (Figure 1D) much more efficiently than after external PCB exposure. Cell morphology was normal and there was no apparent toxicity associated with PCB injection at this concentration. Since the Y276H mutant acts in a constitutive-active manner upon PCB binding, it is also a useful tool to help optimize the requirements for PIF6 recruitment initially without light modulation. In this experiment, Y276H PHYB-CAAX successfully recruited PIF6-EGFP to cell membranes, demonstrating that PCB binding was sufficient to drive PIF6 recruitment. As expected for constitutively active Y276H PHYB-CAAX, this recruitment was not reversible when embryos were exposed to 750 nm wavelength light for 5 min (Figure 1D).

### Reversible Protein Shuttling between the Cytoplasm and Cell Membrane

**Figure 2:**
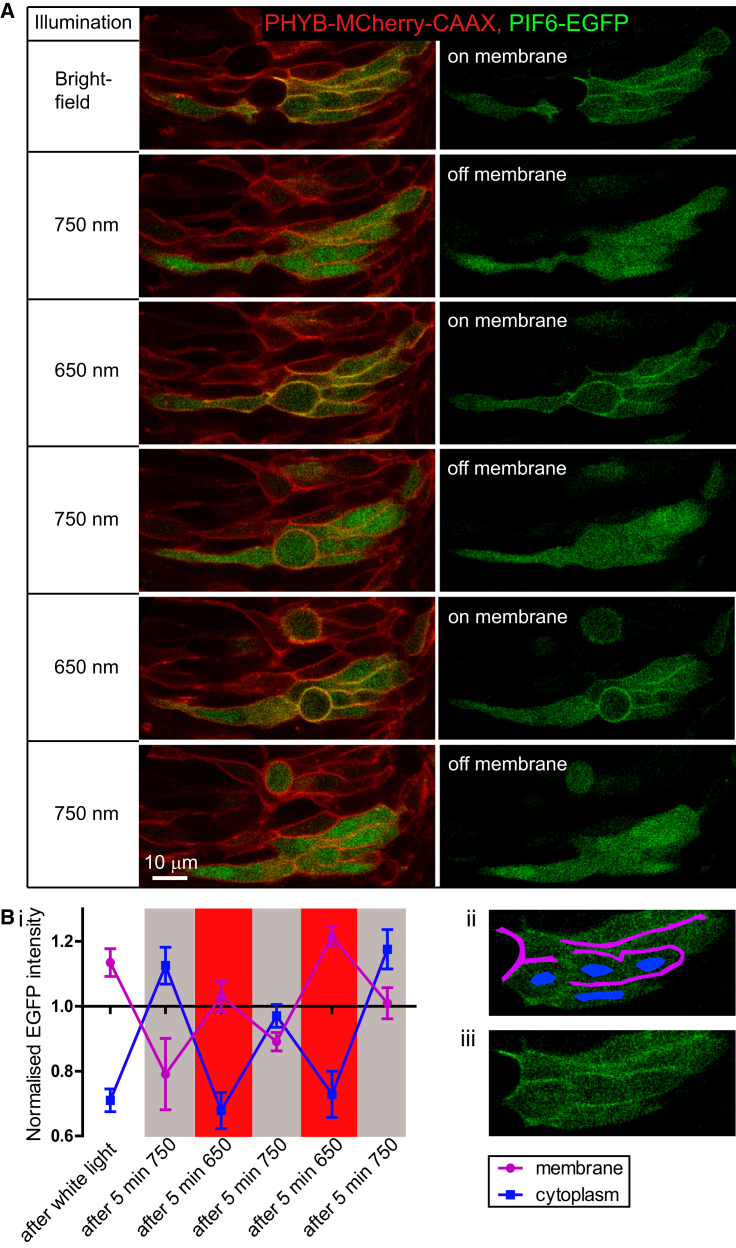
Light-Controlled Shuttling of Protein between Cytoplasm and Membrane In Vivo (A) Sequential images of a horizontal confocal slice through the developing neuroepithelium of a 15-somite embryo labeled with PHYB-MCherry-CAAX and PIF6-EGFP. The embryo was illuminated with alternating 5-min exposures to 650- and 750 nm light. PIF6-EGFP was recruited to the membrane after 650 nm illumination and released from the membrane into the cytoplasm after 750 nm illumination. (B) (i) Relative PIF6-EGFP intensity after alternating 650- and 750 nm illumination, normalized to mean 750 nm levels. Error bars denote SEM. (ii) Illustration of PIF6-EGFP intensity sampling areas in membrane and cytoplasm. (iii) Raw image of (ii). See also related Figure S2.

To test whether PHYB was capable of reversibly recruiting PIF6-tagged proteins to the cell membrane of NE cells within the developing zebrafish embryo brain, embryos mosaically expressing PHYB-MCherry-CAAX and PIF6-EGFP were globally exposed to alternating 5-min intervals of 650 nm (binding is induced; “ON”) and 750 nm (binding is reversed; “OFF”) filtered bright-field (BF) light. The localization of PHYB-MCherry-CAAX and PIF6-EGFP was assessed at the end of each interval. This resulted in the robust and repeatable shuttling of EGFP between the cell membrane and the cytoplasm (Figure 2). The relative differences in EGFP intensity under the different light conditions were quantified (Figure S2), demonstrating a large and significant difference in EGFP intensity between the membrane and cytoplasm when PHYB-PIF6 binding was activated under 650 nm light but a nonsignificant difference under 750 nm light.

**Figure 3:**
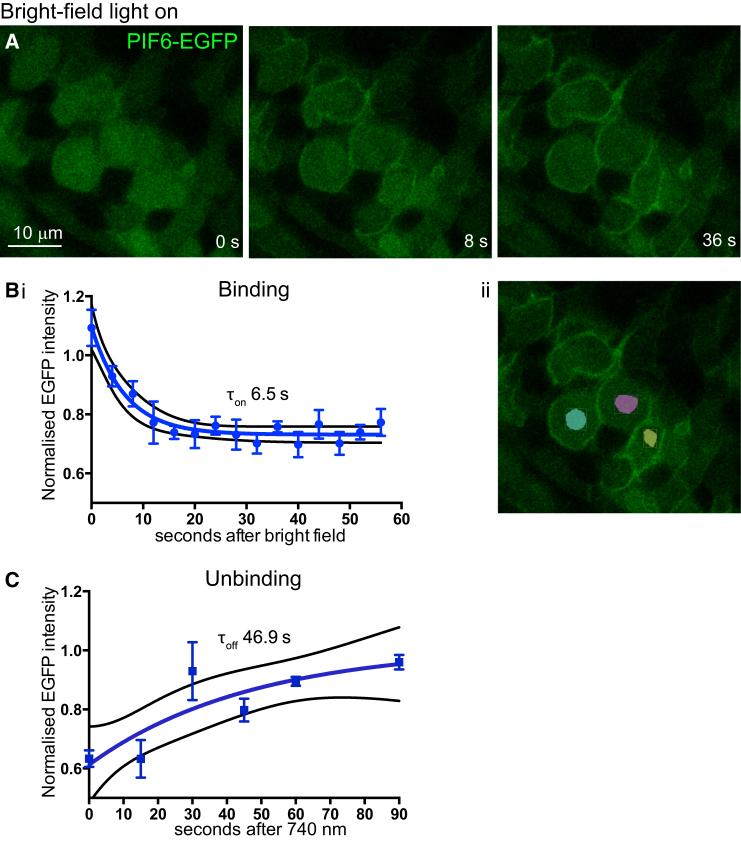
Rapid Kinetics of PHYB-PIF6 Binding and Unbinding (A) Time sequence of a sagittal confocal slice through NE cells of a 15-somite embryo labeled with PHYB-MCherry-CAAX and PIF6-EGFP. Only the PIF6-EGFP signal is shown. At 0 s, 740 nm illumination was replaced with BF illumination and PIF6-EGFP was rapidly recruited to the membrane as illustrated by 8- and 36-s time points. (B and C) Quantification of PIF6-EGFP intensity over time following BF (B) and 740 nm (C) illumination. EGFP intensity was normalized to mean levels at t = 0 s. One-phase exponentials (shown in blue) were fitted to EGFP levels, generating time constants (τ) for binding (B) and unbinding (C) of PIF6 to PHYB. τ = time taken for EGFP levels to either decrease by a factor of 1/e (approximately 36.8% of the original amount) or to increase by a factor of 1 − 1/e (approximately 63.2% of the asymptotic value). Error bars denote SEM. Black lines denote 95% confidence bands. (B) (i) Quantification was from the cells shown in (A). (ii) Illustration of PIF6-EGFP sampling areas. (C) Quantification of unbinding from NE cells in a 22-somite embryo.

To test the speed of PHYB-PIF6 binding and unbinding, we quantified PIF6-EGFP localization over time following BF (ON) and 740 nm (OFF) filtered BF light (Figure 3). This demonstrated that PHYB-PIF6 binding is rapid: a time constant (τ) of 6.5 s was found for decreasing cytoplasmic EGFP levels, which plateaued 33% lower than the starting levels (Figure 3B). Unbinding of PHYB-PIF6 was slower than binding but still relatively rapid: a τ of 46.9 s was found for increasing cytoplasmic EGFP levels (Figure 3C). Cytoplasmic readings were used since they are more robust than membrane readings due to the difficulty of restricting the sampling of fluorescence intensity to membrane alone (discussed later).

Our results demonstrate the efficient phytochrome-mediated reversible movement of proteins between different cellular compartments within a multicellular organism. Rapid time constants show that this system provides a high degree of temporal control.

### Subcellular Control of Protein Localization

**Figure 4:**
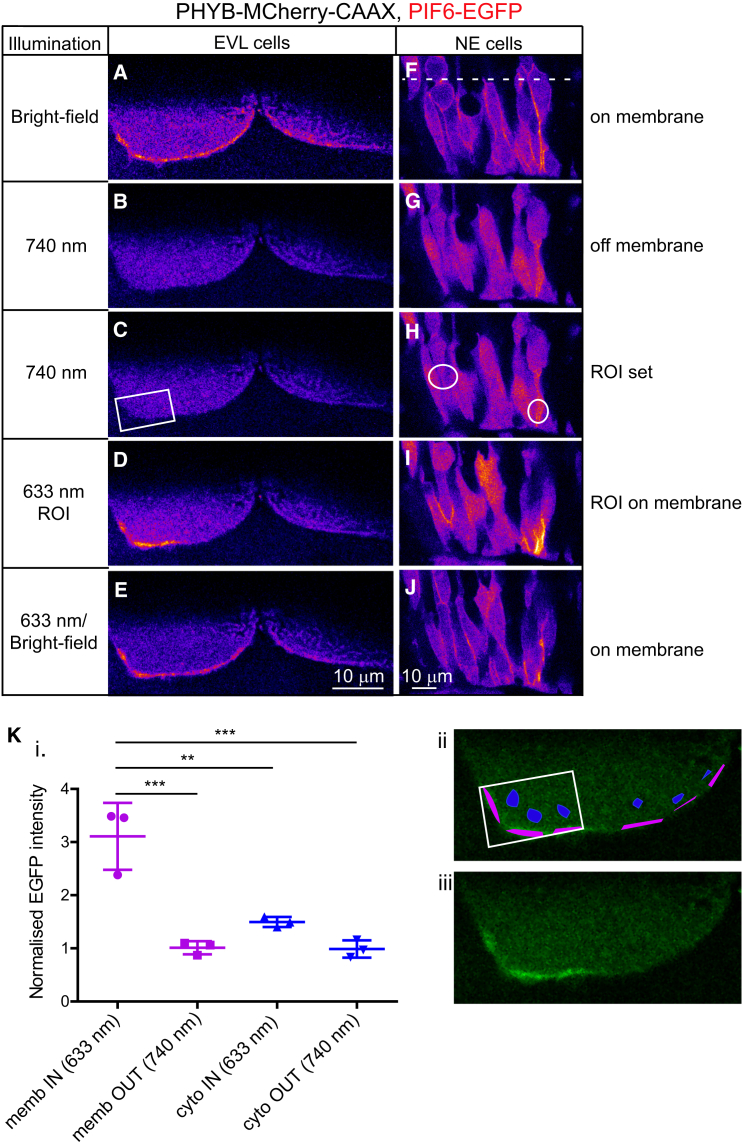
Subcellular Control of Protein Localization (A–J) Sequences of single confocal slices through two EVL cells of a 14-somite embryo (A–E) and a collection of NE cells (F–J). Cells were labeled with PIF6-EGFP and PHYB-MCherry-CAAX. Dotted line denotes developing midline of the neural rod. Only the PIF6-EGFP signal is shown and is pseudo-colored with the Fire look-up table. (A and F) PIF6-EGFP distribution in uniform BF light. (B and G) PIF6-EGFP distribution in uniform 740 nm light for 2 min (EVL) or 4 min (NE). (C and H) Position of ROIs (white rectangle and circles) before 633 nm illumination. (D and I) PIF6-EGFP distribution after 633 nm light was specifically delivered within the ROIs for 30 s (EVL) and 15 min (NE). A uniform background illumination of 740 nm light was also present. PIF6-EGFP was specifically recruited to regions of 633 nm light in three of three EVL cells and eight of eight NE cells. (E and J) PIF6-EGFP distribution following uniform 633 nm light for 1 min (EVL) or BF light for a few seconds (NE). (K) (i) Quantification of PIF6-EGFP intensity inside (IN) and outside (OUT) the 633 nm ROI from the image depicted in (D). EGFP intensity was normalized to mean levels outside the 633 nm ROI. A one-way ANOVA with Tukey’s multiple comparison test was carried out (^∗∗∗^p < 0.001, ^∗∗^p < 0.01). Error bars denote SEM. EGFP intensity at the membrane (memb) inside the 633 nm ROI was significantly higher than all other regions of the cell. EGFP intensities in other regions of the cell were not significantly different to one another. (ii) Illustration of the sample areas of membrane (shown in purple) and cytoplasm (shown in blue) assessed for EGFP intensity inside and outside the 633 nm ROI (white rectangle). (iii) Raw image of (ii).

It is necessary to control protein localization at a subcellular level to probe specific signaling and polarity pathways within cells in vivo. We therefore tested the capability of the phytochrome system to recruit EGFP to specific regions of the plasma membrane in superficial EVL cells and deep NE cells. Embryos mosaically expressing PHYB-MCherry-CAAX and PIF6-EGFP were globally exposed to BF light, causing PIF6-EGFP to be uniformly recruited to cell membranes (Figures 4A and 4F). Embryos were then globally exposed to 740 nm filtered BF light causing PIF6-EGFP to leave the membrane (Figures 4B and 4G). A region of interest (ROI) was then specified over a restricted part of the plasma membrane (Figures 4C and 4H) and low levels of 633 nm laser light were specifically localized to this region in the presence of global 740 nm light. This resulted in the robust and specific recruitment of PIF6-EGFP to the membrane within the ROI (Figures 4D and 4I). Embryos were then globally exposed to low levels of 633 nm laser or BF light, resulting in recruitment of PIF6-EGFP to all cell membranes (Figures 4E and 4J). Subcellular recruitment was rapid, occurring within 30 s of exposure to 633 nm light (Figure 4D). Longer exposures of 15 min to 633 nm light resulted in sustained subcellular recruitment, without obvious bleaching (Figure 4I). Subcellular recruitment was successful both in superficial large EVL cells and in the deeper and smaller NE cells.

To quantify recruitment dynamics, we analyzed the mean EGFP intensity from different areas within the cell depicted in Figure 4D. EGFP intensity in the membrane within the 633 nm ROI was significantly higher when compared with all other regions of the cell (Figure 4K). We also noticed that the restricted subcellular exposure to 633 nm illumination resulted in a relatively larger increase in membrane recruitment when compared with cells receiving a global exposure to 633 nm illumination (compare Figure 4K with Figure S2). This demonstrates that the phytochrome system is an efficient method to accurately, rapidly, and reversibly recruit proteins to specific subcellular regions within a vertebrate embryo.

### Spatiotemporal Control of Pard3 Activity

**Figure 5:**
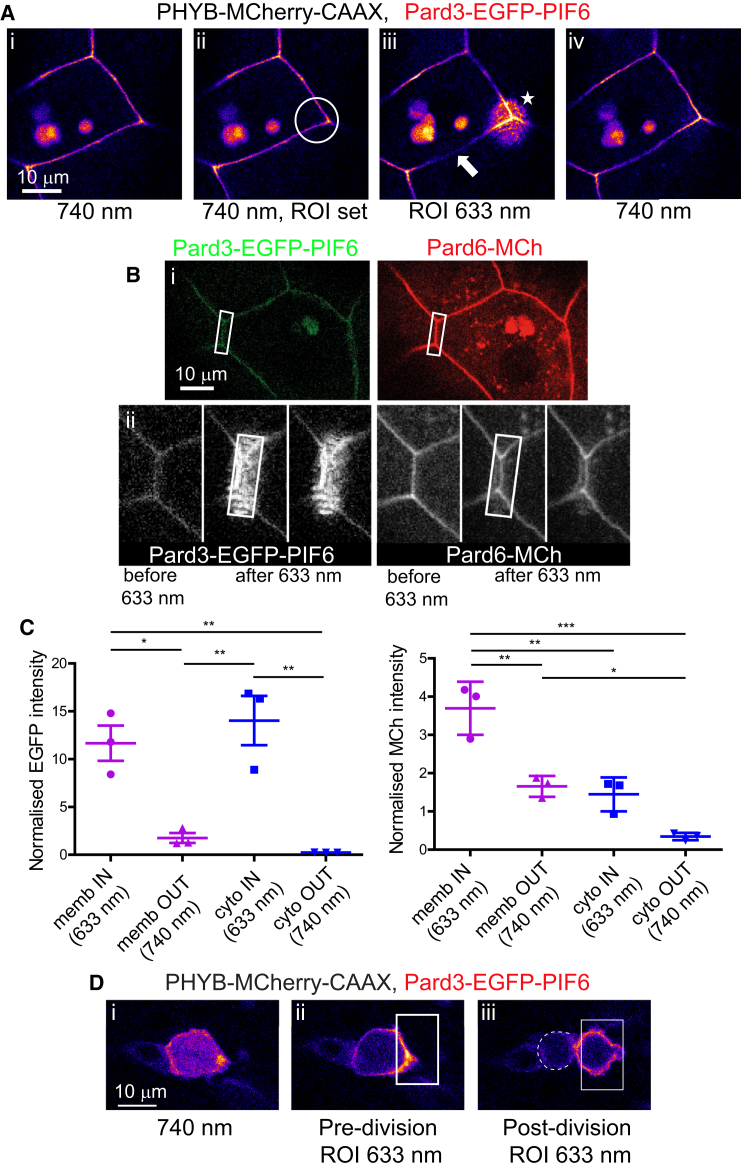
Spatiotemporal Control of Pard3 Activity (A) Single z slice through an EVL cell of an 18-somite embryo, labeled with Pard3-EGFP-PIF6 and PHYB-MCherry-CAAX. The EGFP signal is shown alone and is pseudo-colored with the Fire look-up table. (i) In the unbound state under 740 nm light, Pard3-EGFP-PIF6 is distributed around the whole cell membrane. (ii) Position of ROI (white circle) before 633 nm illumination. (iii) 633 nm light was applied specifically within the ROI with a background uniform 740 nm light for 23 min. Pard3-EGFP-PIF6 specifically accumulated to the ROI (star) and was depleted from the surrounding cell membrane (e.g., arrow). (iv) Uniform 740 nm light for 9 min. Highest levels of Pard3-EGFP-PIF6 were redistributed along the cell membrane. Some intracellular membranes are also labeled with EGFP in these images. (B) (i) Single z slice through the EVL of a 15-somite embryo labeled with Pard3-EGFP-PIF6, PHYB-CAAX, and Pard6-MCherry. The position of the ROI (white rectangle) before 633 nm illumination is shown. (ii) Grayscale images of EGFP and MCh signals showing recruitment of Pard3-EGFP-PIF6 and its partner Pard6-MCh to the ROI (white rectangle) following 633 nm illumination. Areas outside the ROI receive 750 nm light. (C) Quantification of fluorescent intensity differences illustrated in (B) for Pard3-EGFP-PIF6 and Pard6-MCh between membranes (data shown in purple) inside and outside the 633 nm ROI, and between cytoplasm (data shown in blue) inside and outside the ROI. Following 633 nm light illumination, intensity sample areas were placed at the membrane and in the cytoplasm from regions both inside and outside the 633 nm ROI. Fluorescence intensity was normalized to mean levels outside the 633 nm ROI. One-way ANOVAs with Tukey’s multiple comparison tests were carried out (^∗∗∗^p < 0.001, ^∗∗^p < 0.01, ^∗^p < 0.05). Error bars denote SEM. (D) Time lapse of a neural progenitor cell undergoing division within a 13-somite zebrafish embryo neural keel. The cell is expressing Pard3-EGFP-PIF6 and PHYB-MCherry-CAAX, but only the Pard3-EGFP-PIF6 signal is shown and pseudo-colored with the Fire look-up table. (i) Under 740 nm illumination, Pard3-EGFP-PIF6 was distributed around the cell membrane and also expressed in the cytoplasm. (ii) Pard3-EGFP-PIF6 is recruited to the right-hand side of the cell membrane using an ROI (white rectangle) illuminated with 633 nm light for 6 min. (iii) The majority of Pard3-EGFP-PIF6 is inherited into the right-hand daughter following division. Right-hand daughter is within 633 nm ROI (white rectangle), left-hand daughter is outlined by dashed white line. See also related Figure S3.

Because we are interested in understanding the developmental regulation of NE polarity, we next sought to test whether the phytochrome system could be used to alter polarity protein distribution in targeted cells in the embryo. The apical polarity Pard3 is a key protein during epithelial development in many systems (Chen and Zhang, 2013) and localizes gradually but specifically to the tissue midline prior to lumen formation during neural tube development (Buckley et al., 2013, Girdler et al., 2013, Tawk et al., 2007), localizing to apically positioned rings of epithelial junctions (Figures S3A and S3B). Previous work suggests the spatial localization of zebrafish Pard3 may play important roles in establishing NE polarity (Buckley et al., 2013, Girdler et al., 2013, Tawk et al., 2007) and in regulating asymmetric divisions in the neural tube (Alexandre et al., 2010, Dong et al., 2012). We therefore generated a fusion construct of Pard3 with EGFP and PIF6. This Pard3-EGFP-PIF6 fusion protein localized to apical rings in NE cells and EVL cell membranes, similarly to endogenous Pard3 (Figures S3Bii and S3Cii). We then used the PHYB-MCherry-CAAX anchor and 633 nm illuminated ROIs to direct Pard3-EGFP-PIF6 to specific locations (Figure 5). Initially we focused on EVL cells, since they are flat and relatively static. This resulted in the specific enrichment of Pard3-EGFP-PIF6 to the 633 nm ROI and its concurrent depletion from its previous location in the surrounding cell membrane (Figure 5Aiii). Pard3-EGFP-PIF6 enrichment was reversed by ubiquitous 740 nm illumination, which uncoupled the majority of the accumulated Pard3-EGFP-PIF6 from the ROI (Figure 5Aiv).

PIF-tagged proteins are functional in cells in vitro and in unicellular organisms (Levskaya et al., 2009, Toettcher et al., 2011a, Yang et al., 2013). To determine whether Pard3-EGFP-PIF6 in the zebrafish embryo was still able to interact with its common binding partner Pard6, we asked whether a Pard6-MCherry protein would be recruited to the optogenetically enriched domains of Pard3-EGFP-PIF6. We found that when Pard3-EGFP-PIF6 was specifically enriched in a 633 nm ROI, this was followed by a concomitant recruitment of Pard6-MCherry to the ROI (Figure 5B), resulting in significantly more protein at the membrane and cytoplasm inside the ROI than outside (three of four embryos and illustrated in Figure 5C). Increased cytoplasmic recruitment of Pard3-EGFP-PIF6 presumably results because some PHYB-CAAX anchor remains in the cytoplasm rather than the membrane. As a control experiment, we also analyzed whether BFP-ERK2 would be recruited to Pard3-EGFP-PIF6. ERK is not known to associate with Pard3 and no significant increase in BFP-ERK2 intensity was seen within 633 nm ROIs, despite a significantly higher Pard3-EGFP-PIF6 intensity (Figures S3D and S3E). These results demonstrate specific optogenetic enrichment of Pard3 activity and illustrate that the phytochrome system can be used for in vivo manipulation of polarity pathways in a vertebrate embryo.

As a specific example of the potential power of optogenetic PHYB protein localization in developmental biology, we asked whether this system might be able to regulate asymmetric inheritance during neural progenitor mitoses in vivo. Asymmetric inheritance of Pard3 through mitosis is likely to be an important mechanism that regulates daughter cell fate in the vertebrate neural tube (Alexandre et al., 2010, Dong et al., 2012, Kressmann et al., 2015). Therefore we asked whether we could use light to manipulate the inheritance of Pard3. We used a 633 nm ROI to target Pard3-EGFP-PIF6 to the lateral side of NE cells undergoing mitoses during midline crossing divisions (C divisions; Tawk et al., 2007), which have a predictable and stereotyped cleavage orientation (Figure S3A) (Geldmacher-Voss et al., 2003, Tawk et al., 2007). This resulted in the large majority of the Pard3-EGFP-PIF6 construct being inherited by only one of the two daughter cells (Figure 5D). We believe this optogenetic manipulation of protein inheritance illustrates just one exciting possibility of the utility of the phytochrome system for studying fundamental aspects of cell and developmental biology in vivo in multicellular organisms.

## Discussion

We have optimized the expression and delivery of phytochrome components and PCB within living zebrafish embryos and have used the phytochrome system to rapidly and reversibly shuttle proteins between cell membrane and cytoplasm. We also demonstrate specific subcellular recruitment of PIF6-tagged proteins. We manipulate the localization of the polarity protein Pard3 as our proof of principle protein of interest.

### Adapting the Phytochrome System for Use in a Vertebrate Embryo

We achieve functionality of the phytochrome system in vertebrate embryos by solving the challenges of expressing the optogenetic components and delivering the PCB chromophore. *Arabidopsis* PHYB protein is a 1,172-amino acid protein made up of PAS_2, GAF, PHY, PAS, and HKRD domains. Cell culture studies found the HKRD domain of PHYB to be dispensable for reversible PHY-PIF binding but found the PAS repeated domain necessary for allowing reversal of PHY-PIF binding under 750 nm light (Levskaya et al., 2009). However, we found that removal of the C-terminal PAS repeat domain in PHYB markedly improved the expression of PHYB protein in embryos (Figure 1A), and the resulting truncated PHYB was still able to efficiently and reversibly recruit PIF6 protein (Figures 2 and 3). In its native *Arabidopsis*, the C-terminal PAS repeat domain of PHYB protein is necessary for the light-dependent shuttling of PHYB into the nucleus to allow regulation of gene expression (Chen et al., 2005). However, our current study shows the C-terminal signaling domain is not necessary for our purpose. This is in line with earlier observations that the C-terminal is not directly involved in signal transduction and that, if made to localize in the nucleus, the isolated N terminus mediates more sensitive signaling responses than full-length PHYB (Matsushita et al., 2003). Studies in yeast also found that an N-terminal PHYB, comprising residues 1–621, had a greater activity with PIF proteins than full-length PHYB (Pathak et al., 2014).

In addition to the PHYB and PIF anchor and bait proteins, heterodimerization also requires the presence of the chromophore PCB. We achieved this by co-injection of HPLC-purified PCB protein (Figure S1 and Supplemental Experimental Procedures) along with RNA encoding PHYB and PIF6 proteins directly into one cell of 16- to 32-cell stage embryos. This results in a mosaic distribution of the relevant proteins throughout the embryo without apparent cell toxicity and is more efficient than bathing the whole embryo in PCB (Figure 1D). In the future, more uniform levels of PCB could be achieved throughout the zebrafish by generating a transgenic line with the two genes necessary for PCB synthesis (heme oxygenase 1 [HO1] and PCB:ferredoxin oxidoreductase [PcyA]). This would also help combat any dilutional effects that may be seen at later developmental time points. This has been achieved in bacteria (Levskaya et al., 2005) and in mammalian cells in vitro (Muller et al., 2013) but has so far not been attempted in whole multicellular organisms. However, we have found that, at least up to 24 hpf, injection of PCB protein is sufficient for optogenetic experiments, and the transgenic generation of PCB is not required.

### Efficiency of PHYB-PIF6 Binding/Unbinding In Vivo

The efficiency of PHYB-PIF6 binding and unbinding achieved in vivo within the embryo was high. The speed of binding was only slightly slower than in cell culture, with τ = 6.5 s for cytoplasmic depletion during PHYB-MCherry-CAAX:PIF6-EGFP binding in zebrafish (Figure 3B), compared with 1.3 s during PHYB-MCherry-CAAX:PIF6-YFP binding in NIH3T3 cells (Levskaya et al., 2009). This allowed us to reproduce the rapid and reversible shuttling of fluorescent protein between the cytoplasm and membrane as seen in cell culture (Figures 2 and 3). Relatively higher levels of membrane recruitment were achievable when binding illumination was restricted to subcellular ROIs (compare Figure 4K with Figure S2). Unbinding speed was slower than binding, with τ = 46.9 s for cytoplasmic increase in EGFP levels (Figure 3C). This may partly be due to the differences in method used for measuring τ_on_ and τ_off_ rates (see Experimental Procedures). However, we suggest that the main reason is that even ambient light can cause low levels of PHYB-PIF6 binding, therefore slowing down unbinding rates under 740 nm light. Conversely, only wavelengths of light very close to 740–750 nm were sufficient to initiate unbinding. This sensitivity necessitates careful experimental design and control but is also advantageous since it is highly unlikely that PHY-PIF6 binding would be reversed by exposure to ambient light.

Quantifying membrane fluorescence in the embryo has two difficulties. The first is that cell shapes are dynamic, and re-sampling a precise region of membrane over time is subject to some error. Second, it was difficult to eliminate all adjacent cytoplasmic fluorescence from our membrane readings, which would artificially slow the calculated time constants and increase their variance. For these reasons, we suspect that the cytoplasmic readings depicted in Figure 3 reflect truer kinetic measurements for our experiments.

### Subcellular, Spatiotemporal Control of Pard3 Activity in Live Zebrafish Embryos

To demonstrate that the phytochrome system can be used to alter polarity pathways within the zebrafish brain, we chose to manipulate Pard3 localization during NE development. We are interested in understanding the development and importance of NE polarity, and Pard3 is a key apical polarity protein in epithelia and has been shown to be instrumental in zebrafish neural tube development. For example, it is important in mediating mirror-symmetric cell division during neural rod development (Tawk et al., 2007), allowing proper lumen formation (Buckley et al., 2013), positioning centrosomes (Hong et al., 2010), and promoting neurogenic divisions (Alexandre et al., 2010). Global knockout of Pard3 can be informative for some experimental aims, but a targeted manipulation of Pard3 localization should elucidate the importance of Pard3 in organizing the subcellular polarization events during neural development. We have demonstrated that the phytochrome system can be used to achieve this goal by optogenetically altering subcellular Pard3-EGFP-PIF6 distribution (Figure 5). This consequently also altered the subcellular distribution of the partner protein Pard6-MCherry (Figures 5B and 5C), illustrating phytochrome-mediated spatiotemporal control of Pard3 activity. By manipulating the location of Pard3 and other proteins in the developing neural primordium, we now hope to determine how protein localization is able to direct the pattern of epithelialization and lumen formation in vivo (Buckley et al., 2013, Girdler et al., 2013). We also hope to use our ability to manipulate protein inheritance during mitoses (Figure 5D) to directly test their suggested roles in asymmetric divisions (Alexandre et al., 2010, Dong et al., 2012, Kressmann et al., 2015).

### Limitations

Having established a proof of principle for using the phytochrome system for specific spatiotemporal control of target protein activity, we would like to suggest some improvements to our basic technique for future exploration.

First, the PHYB-PIF6 system does not eliminate the endogenous mechanisms that determine normal protein localization (in the case of Pard3, those mechanisms that drive it to the epithelial junctional belts at the interface of apical and basolateral domains). Thus, PHYB-PIF6-driven ectopic localization will be subject to competition from endogenous localization signals. The creation of ectopic foci of polarity proteins should be informative in determining the particular contributions of these proteins for establishing the cells' organization. However, a genetic approach to replace endogenous proteins with PIF- and PHYB-tagged versions will eliminate the endogenous pool of untagged protein that would otherwise be inert to optogenetic manipulations. In this way even protein targeted to a particular cell location by endogenous mechanisms will be subject to the competitive influence of the optogenetic manipulation, therefore strengthening the experimental phenotype. Generating stable transgenic lines of zebrafish would also combat the variance in levels of fusion protein expression that result from transient techniques such as RNA injections. Standardizing protein expression levels is important since we found that a relatively higher level of PHYB is necessary to robustly recruit PIF6 protein to the membrane and to deplete it from the cytoplasm. This is consistent with observations in cell culture, which showed an appropriate ratio of PHYB and PIF protein inside an individual cell is critical in assaying PHYB-PIF interactions (Toettcher et al., 2011a). A more sophisticated modulation of recruitment levels, even when there is cell-cell variability in protein levels, could be achieved by using a computational feedback controller, as shown in cell culture (Toettcher et al., 2011b).

A second improvement would be to use a more sophisticated method to target the 633 nm and 750 nm wavelengths to particular parts of the cells. Specifying an ROI using a point scanner approach, as in Figures 4 and 5, is a simple and effective way in which to pattern light illumination at a single z level. However, the cone effect of conventional confocal microscopes will also affect binding out of the targeted plane. In addition, complementary ROIs of 633 nm binding light and 740 nm unbinding light would be advantageous for precise and efficient spatial control. The use of spatial light modulation devices (Lutz et al., 2008) would help with these approaches.

### Conclusions

We have successfully developed the use of light-induced heterodimerization to modify subcellular protein localization within the zebrafish embryo using the phytochrome system. This system is rapidly reversible and allows a very high level of temporal and spatial control, with binding rates close to those seen in cell culture.

As a proof of principle, we use the phytochrome system to spatiotemporally manipulate the localization of the polarity protein Pard3 at a subcellular level in targeted cells within a whole embryo, with corresponding relocalization of the partner protein Pard6. This approach to target protein localization will complement other experimental approaches to understand in vivo function such as knockouts or functional abrogation. The level of experimental control afforded by the phytochrome heterodimerization technique will be particularly useful to studies in which protein localization and timing is critical, such as studies of cell polarity and migration. We envision that the specific optimizations and general methodology detailed here will enable the similar use of the phytochrome system to reversibly control subcellular protein localization in other multicellular organisms.

## Experimental Procedures

All procedures were carried out with Home Office approval and were subject to local Ethical Committee review.

### Far-Red and Infrared Light Sources

Whole-embryo illumination experiments were carried out under bright light filtered through a 650 nm band-pass filter (Edmund Optics) and a 750 nm long-pass filter (Newport, RG9). Subcellular recruitment experiments were carried out using a low intensity 633 nm laser on a laser scanning confocal microscope (LSM; Leica SP5) within an ROI. The background was illuminated with filtered 740 nm light from a Schott KL 1500 LCD light source, filtered with band-pass glass (Envin Scientific). When taking confocal images of EGFP localization during subcellular experiments, it was necessary to briefly switch off both the 633 nm laser and the 740 nm unbinding light.

### PCB

For bath application, 300 μM HPLC-purified PCB (see Supplemental Experimental Procedures) was added to embryo medium overnight. For injection, 1.5 pM HPLC-purified PCB was injected into one cell of a 4- to 32-cell stage embryo, along with RNA encoding the relevant fusion proteins.

### Binding and Unbinding Assays

Binding and unbinding assays were carried out on an LSM (Leica SP5). For the binding assay (Figure 3B), we replaced 740 nm light with BF light at 0 s and took an image under 488 nm light every 4 s. Since even 488 nm light caused binding in our experiments, we took a different approach for the unbinding assay (Figure 3C). At 0 s we replaced BF light with 740 nm light and took an image under 488 nm light after 15 s. We then recruited GFP to the membrane under BF light for approximately 1 min before again replacing BF light with 740 nm light and taking an image under 488 nm light after 30 s. This process was repeated for all time points assessed.

### Image Analysis

To calculate relative EGFP levels, EGFP intensity readings were calculated from three to four intensity sample areas from both the areas of interest and background. The mean background reading was subtracted from each of the sample area readings. Sample area readings were then normalized to overall EGFP levels, as described in the figure legends. Statistical analyses are described in the figure legends and were carried out using GraphPad Prism software (^∗∗∗^p < 0.001, ^∗∗^p < 0.01, ^∗^p < 0.05).

Also see Supplemental Experimental Procedures.

## Author Contributions

C.E.B. carried out most of the experimental work and co-wrote the manuscript. R.E.M. carried out the experimental work for Figures 5B, 5C, S3D, and S3E. A.R. carried out initial zebrafish phytochrome work, helped with experimental work, and provided Figure S1. A.R.G. helped with experimental work and made the shortened N-PAS2-GAF-PHY version of PHYB. O.D.W. and J.D.W.C. oversaw the work and co-wrote the manuscript.

## Supplemental Information

Document S1. Supplemental Experimental Procedures and Figures S1–S3

Document S2. Article plus Supplemental Information
