# Supplementary material for: Reversible Optogenetic Control of Subcellular Protein Localization in a Live Vertebrate Embryo
Source: Dev Cell. 2016 Jan 11;36(1):117–26. doi: 10.1016/j.devcel.2015.12.011 (PMC4712025; doi:10.1016/j.devcel.2015.12.011)
Supplement: Document S1. Supplemental Experimental Procedures and Figures S1–S3 [file mmc1.pdf]

Developmental Cell, Volume *36*

Supplemental Information

Reversible Optogenetic Control of Subcellular Protein Localization in a Live Vertebrate Embryo

Clare E. Buckley, Rachel E. Moore, Anna Reade, Anna R. Goldberg, Orion D. Weiner, and Jonathan D.W. Clarke

Supplemental Inventory

Figure S1, related to Figure 1: PCB toxicity.
Illustrates the low toxicity of HPLC purified PCB, first introduced in Figure 1, and used throughout the study.

Figure S2, related to Figure 2: Statistical analysis of EGFP intensity
The relative differences in EGFP intensity under the different light conditions depicted in Ffigure 2 are quantified here.

Figure S3, related to Figure 5: Pard3 localisation and BFP-ERK2 negative control
Illustrates the localisation of the Pard3-EGFP-PIF6 used in Figure 5 in comparison with endogenous Pard3 expression. Also depicts a negative control for the experiment in Figure 5B and C.

Supplemental Experimental Procedures
Includes details of the fusion constructs used and the HPLC purification protocol for PCB.

Supplemental References

Figure S1, related to Figure 1: PCB toxicity

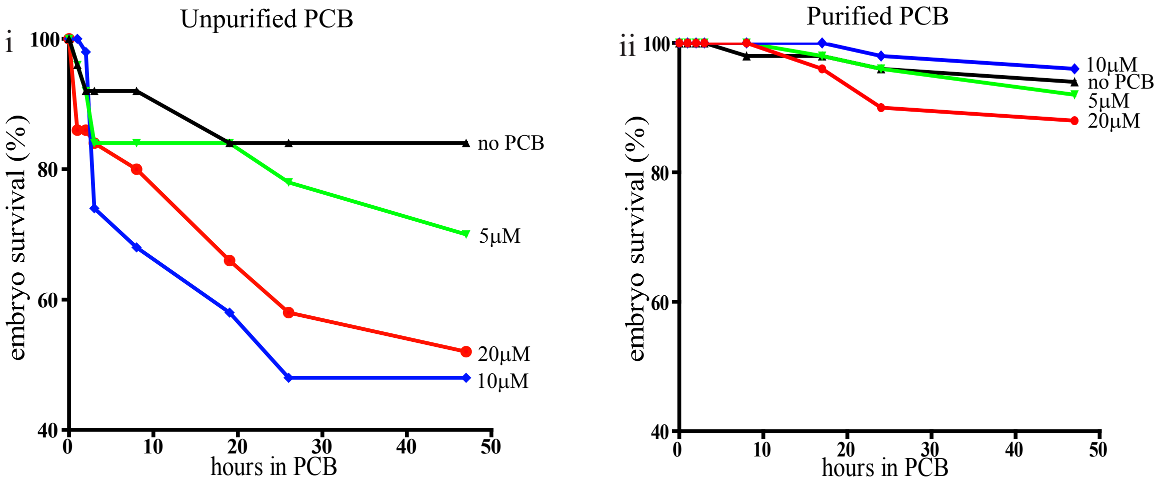

Time-course of percentage embryo survival after 3-5h.p.f. embryos were dechorionated and bathed in increasing concentrations of (i) unpurified PCB and (ii) HPLC-purified PCB. 50 embryos were used for each concentration. Embryo survival was markedly greater in purified PCB.

Figure S2, related to Figure 2:
Statistical analysis of EGFP intensity

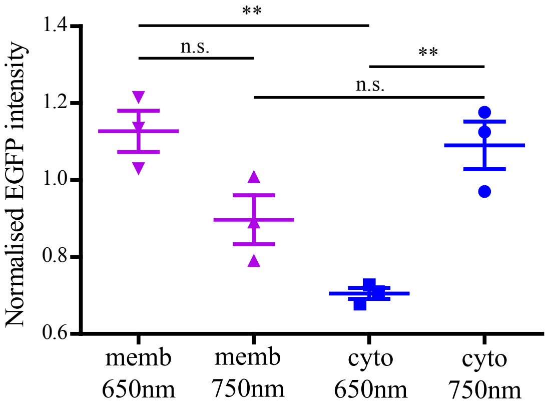

The mean EGFP intensity readings from membrane and cytoplasmic sample areas in each of the images from Figure 2B were used to compare the mean EGFP intensity under different light conditions and at different regions of the cell. One-way ANOVA with Tukey's multiple comparison test was carried out. Error bars denote standard error of the mean.

Figure S3, related to Figure 5: Pard3 localisation and BFP-ERK2 negative control

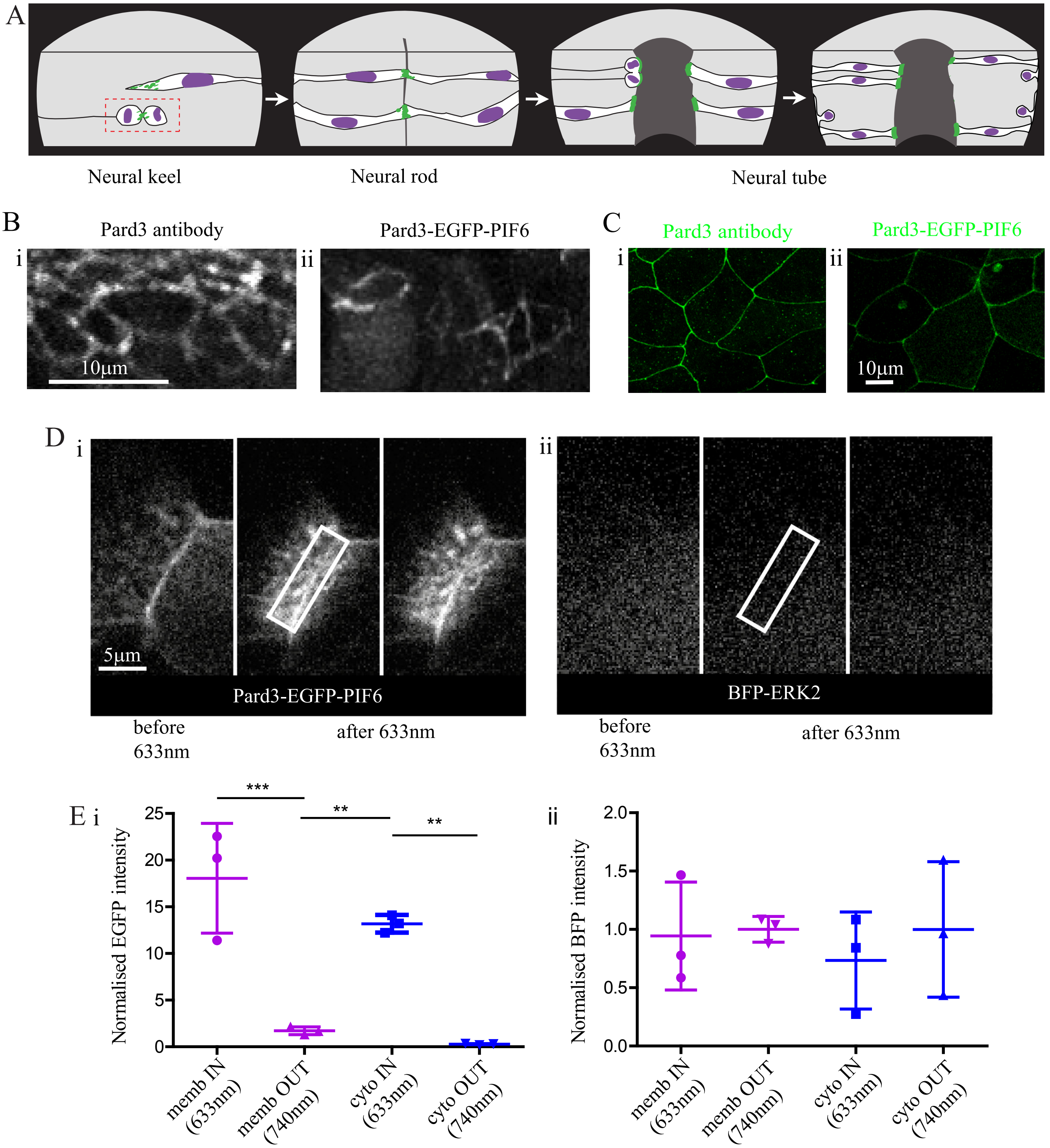

A. Cartoon illustrating the localisation of Pard3 (green) during the maturation of neuroepithelial cells within the developing neural tube. Red dotted box shows the region imaged in figure 5D. At keel stages Pard3 broadly localises to the tissue centre before division. Midline crossing divisions (C-divisions) occur at the centre of the tissue and localise Pard3 more specifically to their cleavage planes, helping to produce a precise localisation of Pard3 at the tissue midline at rod stages. The neural tube lumen then cavitates from this midline, resulting in the opening of the neural tube. Later, NE cells undergo further rounds of division at the Pard3-labelled apical surface, resulting in the production of both NE cells and neurons. **B.** En face images of the neuroepithelial apical surface labelled with anti-Pard3 antibody or Pard3-EGFP-PIF6. **C.** En face images of EVL cells labelled with anti-Pard3 antibody or Pard3-EGFP-PIF6. Pard3 is present at membranes around the perimeter of cells. A small amount of fusion protein is also present in intracellular membrane inclusions. **D.** Single confocal slice through the EVL of an embryo labelled with Pard3-EGFP-PIF6, PHYB-CAAX and BFP-ERK2. Signal for Pard3-EGFP-PIF6, PHYB-CAAX and BFP-ERK2 are shown in (i) and (ii) respectively. Pard3-EGFP-PIF6 is robustly recruited to the 633nm ROI, but there is no concomitant recruitment of BFP-ERK2 to this region. **E.** Normalised fluorescence intensities for Pard3-EGFP-PIF6 and BFP-ERK2 from experiment shown in (D). One-way ANOVAs with Tukey's multiple comparison tests were carried out. Error bars denote standard error of the mean.The experiment was repeated 5 times and a significant increase in membrane intensity within the 633nm ROI when compared with membrane regions in the same cells outside the ROI was seen in 5/5 cases for EGFP, but 0/5 cases for BFP. In 2/5 cases there was also a significant increase in EGFP intensity within the cytoplasm in the 633nm ROI, but no concomitant increase in cytoplasmic BFP-ERK2.

Supplemental Experimental Procedures

Embryo Care
Embryos were collected, staged and cultured according to standard protocols (Kimmel et al., 1995; Westerfield, 2000). All procedures were carried out with Home Office approval and were subject to local Ethical Committee review.

Embryo Injections
Embryos were injected using standard injection protocols (Westerfield, 2000). For ubiquitous distribution of mRNA, embryos were injected at a 1 to 4-cell stage. Embryos were mosaically labelled by injecting mRNA into one blastomere of a 16-32 cell stage embryo.

Fusion constructs
Fusion constructs were made using the PCS2+ plasmid backbone using an enzymatic assembly method (Gibson et al., 2009). 10 amino acid polyglycine-serine linkers were used in most constructs. RNA was then synthesised with the Ambion mMessage mMachine System from the SP6 promoter (AM1340). Details of constructs are below.

Construct details

Construct name	Composition	Approximate amount injected
1. N-PAS2-GAF-PHY-PAS	1-917 amino acids of Arabidopsis PHYB (Gene ID: 816394), tagged with MCherry fluorescence protein and the CAAX membrane moiety. Mammalian codon optimised	0.2 ng
2. N-PAS2-GAF-PHY/PHYB-MCherry-CAAX	1-621 amino acids of Arabidopsis PHYB (Gene ID: 816394), tagged with MCherry fluorescence protein and the CAAX membrane moiety. Mammalian codon optimised	0.2 ng-0.25 ng
3. Y276H PHYB-CAAX and Y276H PHYB	1-621 amino acids of Arabidopsis PHYB (as in construct 2) with and without the CAAX membrane moiety tag. Tyrosine residue 276 of PHYB was mutated to Histidine.	0.2 ng-0.25 ng (CAAX tagged)<br>0.15 ng (without CAAX tag)
4. H2A-GFP	Histone 2A tagged with GFP	0.04 ng
5. PIF6-EGFP	1-100 amino acids of Arabidopsis PIF6 (Gene ID: 825382), tagged with EGFP fluorescent protein. Mammalian codon optimised.	0.05 ng
6. Pard3-EGFP-PIF6	Full length zebrafish Pard3 (Gene ID: 403050), tagged with EGFP fluorescent protein and Arabidopsis PIF6 (as in construct 5).	0.1-0.15 ng
7. Pard6beta-mCherry	Full length Pard6beta (Gene ID: 100124652) tagged with mCherry fluorescence protein.	0.25 ng
8. BFP-ERK	Erk2 tagged with BFP fluorescence protein.	0.13 ng

Pard3 Immunohistochemistry
Embryos were fixed for 3 hours in DENTs fixative (80% Methanol, 20% DMSO) then rehydrated and

stained over night in Rabbit-anti-Pard3 (Millipore 070-330) at 1:100 dilution at 4°C. They were then rinsed in PBT and stained with Alexa goat-anti-rabbit 488 (Invitrogen A11034) at 1:500 dilution for several hours at r.t.p.

Confocal imaging
Embryos were mounted in low melting point agarose and imaged on upright Leica LSM microscopes (SP5 and SP8) using high Na water dipping x25 and x65 objectives. Images were processed using Volocity and Image J software.

Phycocyanobilin (PCB) extraction and HPLC purification
We found that the standard crude PCB obtained from Spirulina (Holmes, 1984; Levskaya et al., 2009) proved phototoxic to the embryo even when delivered at concentrations as low as 10 μM (Figure S1i). However, HPLC purification of PCB greatly reduced this toxicity (Figure S1ii). PCB was extracted as described previously (Toettcher et al., 2011). The PCB was then further purified: The PCB-DMSO solution was spun at 5000xG for 10 minutes at room temp. The supernatant was passed through glass wool and a 0.45 μm filter. The filtered PCR-DMSO solution was run on a C18 column (Waters Atlantis Prep T3 OBD 5μm, 19x100mm column). Solution A: 0.5% formic acid in water. Solution B: 0.5% formic acid in acetonitrile. The sample was run with a gradient to 5% solution B in 5 minutes, followed by a gradient to 100% solution B in 11 minutes. Methanol-extracted PCB mixture produced several peaks (depicted by the hand-drawn example below).

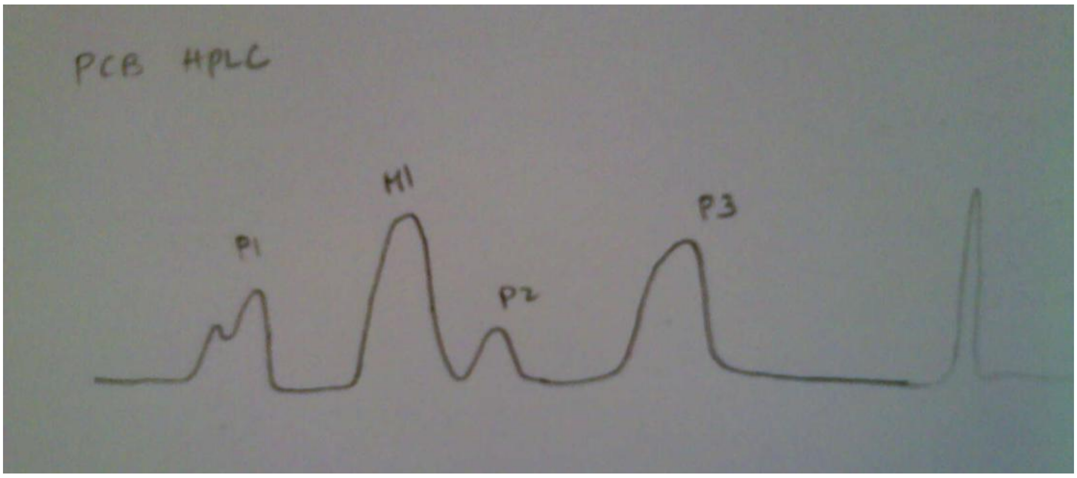

Liquid chromatography-mass spectrometry confirmed that only the major peak (M1) contained PCB (587.6 g/mol). PCB from the major peak was pooled, lyophilized and resuspended in a minimal amount of DMSO. To obtain the final PCB concentration, a small amount of the final preparation was diluted 1:100 into 1 mL 95:5% MeOH:HCl (37.5%) solution and the absorbance at 680 nm measured. The concentration in mM is calculated as A680 x 2.64. In 95:5% MeOH:HCl, the extinction coefficient of PCB is 37,900 M^{-1} cm^{-1}. Typical final concentrations from this procedure were between 3 and 15 mM. Wavelength absorbance scans showed absorbance peaks at 340 nm and 680 nm (shown below).

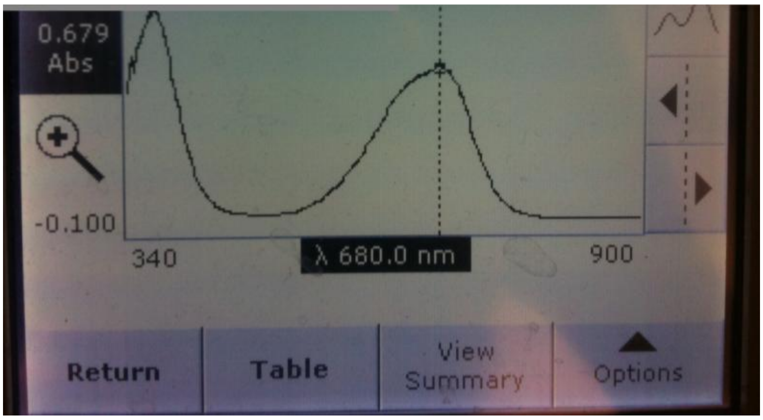

There is another non-functional fraction which also has this absorbance reading. This has a molecular

weight of 601.7 g/mol from analytical LCMS from Peak 3. The PCB was diluted to 12.5 mM in DMSO and stored at -20 °C until use.

Supplemental References

Gibson, D.G., Young, L., Chuang, R.Y., Venter, J.C., Hutchison, C.A., 3rd, and Smith, H.O. (2009). Enzymatic assembly of DNA molecules up to several hundred kilobases. Nature methods *6*, 343-345.

Holmes, M.G. (1984). Techniques in Photomorphogenesis (Academic Press, Inc. (London) Ltd.).

Kimmel, C.B., Ballard, W.W., Kimmel, S.R., Ullmann, B., and Schilling, T.F. (1995). Stages of embryonic development of the zebrafish. Dev Dyn *203*, 253-310.

Westerfield, M. (2000). The zebrafish book. A guide for the laboratory use of zebrafish (Danio rerio), 4th ed. edn (Eugene: Univ. of Oregon Press).
